# Modeling Users' Cognitive Performance Using Digital Pen Features

**DOI:** 10.3389/frai.2022.787179

**Published:** 2022-05-03

**Authors:** Alexander Prange, Daniel Sonntag

**Affiliations:** ^1^German Research Center for Artificial Intelligence (DFKI), Saarland Informatics Campus, Saarbrücken, Germany; ^2^Applied Artificial Intelligence, Oldenburg University, Oldenburg, Germany

**Keywords:** digital pen features, machine learning, cognitive assessments, neuropsychological testing, Clock Drawing Test, Trail Making Test, Rey-Osterrieth Complex Figure Test, deep learning

## Abstract

Digital pen features model characteristics of sketches and user behavior, and can be used for various supervised machine learning (ML) applications, such as multi-stroke sketch recognition and user modeling. In this work, we use a state-of-the-art set of more than 170 digital pen features, which we implement and make publicly available. The feature set is evaluated in the use case of analyzing paper-pencil-based neurocognitive assessments in the medical domain. Most cognitive assessments, for dementia screening for example, are conducted with a pen on normal paper. We record these tests with a digital pen as part of a new interactive cognitive assessment tool with automatic analysis of pen input. The physician can, first, observe the sketching process in real-time on a mobile tablet, e.g., in telemedicine settings or to follow Covid-19 distancing regulations. Second, the results of an automatic test analysis are presented to the physician in real-time, thereby reducing manual scoring effort and producing objective reports. As part of our evaluation we examine how accurately different feature-based, supervised ML models can automatically score cognitive tests, with and without semantic content analysis. A series of ML-based sketch recognition experiments is conducted, evaluating 10 modern off-the-shelf ML classifiers (i.e., SVMs, Deep Learning, etc.) on a sketch data set which we recorded with 40 subjects from a geriatrics daycare clinic. In addition, an automated ML approach (AutoML) is explored for fine-tuning and optimizing classification performance on the data set, achieving superior recognition accuracies. Using standard ML techniques our feature set outperforms all previous approaches on the cognitive tests considered, i.e., the Clock Drawing Test, the Rey-Osterrieth Complex Figure Test, and the Trail Making Test, by automatically scoring cognitive tests with up to 87.5% accuracy in a binary classification task.

## 1. Introduction

Neurocognitive testing is a noninvasive method for measuring brain function by evaluating specific cognitive abilities, including memory, fine motor control, reasoning, and recognition. Despite recent technological advances, the majority of cognitive assessments used in practice is still conducted using pen and paper with manual scoring by the physician afterwards. This includes verbal interview-like tests, in which the physician takes notes of the patient's answers, as well as tests in which the patient is asked to write or sketch as part of the assessment task. Cognitive assessments have been the subject of recent debate because there are limitations when they are conducted using pen and paper. For example, the collected material is monomodal (written form) and there is no direct digitalization for further and automatic processing. In addition, the results can be biased depending on the physician's level of expertise.

Nowadays, commercially available devices, such as styluses, digital pens and digitizer tablets are capable of recording pen input in real-time. The resulting data is a continuous stream of timestamped x/y coordinates. Various, mathematically defined, geometrical, spatial, temporal, pressure and other features can be extracted directly from the raw input stream. These digital pen features can then be used as input to train supervised machine learning (ML) algorithms to automatically score cognitive assessments, which has several benefits. For example, automatic assessments are potentially more objective than human assessments, because they are not biased by the physician's level of expertise. They can include the analysis of new features (such as small movements not visible to the naked eye) and allow physicians to shift their attention to other behavioral cues such as speech or facial expressions. In addition, modern hospital processes require a direct digitization of test results without time-consuming and expensive manual transcription.

We present an interactive cognitive assessment tool that records user input using a digital pen and automatically scores the test by analyzing a set of features extracted directly from the pen input. The clinician can observe the sketching process in real-time on a mobile tablet, which opens up new opportunities for telemedicine settings and eases compliance with Covid-19 distancing regulations. Patients could, for instance, perform cognitive assessments from home or behind a protective barrier in the clinic. Afterwards, the results of our automatic test analysis are presented in real-time to the clinician, thereby reducing manual scoring effort and producing more objective reports.

Several automated scoring systems based on multi-stroke sketch recognition and machine learning have been published previously (Canham et al., 2000; Davis et al., 2015; Niemann et al., 2018; Prange and Sonntag, 2019; Prange et al., 2019). Such systems typically score assessments based on their scoring schemes, thereby simulating the work of a clinician, and need to include task dependent knowledge for each type of assessment. This process is time-consuming and expensive, as enough annotated data needs to be available and each cognitive assessment needs to be modeled and implemented individually. In the present work we go one step further. Instead of performing traditional task dependent content analysis, we consider only the sketch characteristics, which we extract from the raw pen input signal using a feature set of more than 170 digital pen features. This approach allows us to generalize the classification of cognitive performance independently of the considered task and opens up new approaches for transparent behavior analysis in pen-based intelligent user interfaces.

Using our interactive cognitive assessment tool, we record a total of 240 samples of the CDT, TMT and ROCF. Based on the scoring results we label each sample as either healthy or suspicious, latter indicating the need for further testing or intervention. We use this as the ground truth and use the feature vectors as input for 10 machine learning methods, with the goal to classify cognitive test performance solely based on the sketch characteristics. Our aim is to support fully automatic, objective and accurate diagnostics of pen sensor input, which can be used in hospitals and retirement homes to transparently evaluate cognitive performance (i.e., without explicit testing), to guide medical interventions, and to adapt cognitive training in a personalized manner.

Two approaches for the automatic analysis of paper-pencil-based cognitive assessments are presented here as part of our interactive cognitive assessment tool. The first one is a traditional approach that uses digital pen features to perform a content analysis of drawn sketches and scores the test based on the predefined medical scoring scheme, thereby automating the process that is normally conducted manually by a physician. The next big step in analyzing digital cognitive assessments is to predict cognitive performance independently of the test content, by looking only at the writing and sketching behavior of users, which is explored in the second approach. In this approach the cognitive test performance is predicted by only considering the digital pen features, which are applicable independent of the task, without performing further content analysis. The aim is to support more automatic, more objective and accurate diagnostics of pen sensor input, which can be used in hospitals and retirement homes to transparently evaluate cognitive performance (i.e., without explicit testing), to guide medical interventions, and to adapt cognitive training in a personalized manner.

## 2. Related Work

Our related work is structured into three sections. The first section summarizes digital pen feature sets, and the second section presents most commonly used cognitive assessments. Finally, in the third section we explore different approaches for analyzing cognitive assessments using digital pen features.

### 2.1. Digital Pen Feature Sets

Traditionally, stroke level features are most often used for statistical gesture recognition. One of the most prominent sets of pen features was presented by Rubine (1991). It contains a total of 13 features that are designed to reflect the visual appearance of strokes in order to be used in a gesture recognizer. The feature set includes geometric features, such as cosine and sine values of the initial angle of the gesture, the length of the gesture or total angle traversed, and temporal aspects, such as maximum speed and duration of gestures. According to the author, these features were determined empirically to work well on several different gesture sets. Applying linear classifiers to these gesture sets, Rubine reports recognition rates of over 96%, even for relatively small training set sizes of 15 samples per class. Rubine's feature set has been successfully applied in pen-based intelligent user interfaces (Stapleton et al., 2015; Williford et al., 2020), multi-touch gesture recognition (Cirelli and Nakamura, 2014; Rekik et al., 2014) and even eye-tracking analysis (Çağla Çğ and Metin Sezgin, 2015; Alamudun et al., 2017).

More recent work by Willems and Niels (2008) defines a total of 89 features using formal mathematical descriptions and algorithms. While their technical report mainly focuses on the implementation details, their other publications show ML-based applications of their feature set for multi-stroke gesture recognition (Willems et al., 2005, 2009; Willems and Vuurpijl, 2007), forensic writer identification (Niels et al., 2007) and writer verification (Brink et al., 2011). The Willems and Niels feature set includes several features, which are also present in Rubine's set (Rubine, 1991), but it is overall comprised of computationally more complex features. These include curvature, perpendicularity, complex hull properties, histogram analysis, acceleration and many more. It is also one of the few feature sets that includes force-based characteristics, which model the pressure that users apply to the writing surface, and which, depending on the hardware device, is either measured directly by the digital pen or the digitizer tablet. In addition, the Willems and Niels feature set contains higher level *meta features*, which account for crossings, connected strokes and straight lines.

In 2013, Adrien Delaye and Eric Anquetil introduced the HBF49 (Heterogeneous Baseline Feature) set (Delaye and Anquetil, 2013), which contains 49 features and is specifically designed to be used as a reference for the evaluation of symbol recognition systems. Similar to Rubine (1991) an empirical constructive approach is adopted for designing this feature set, with the aim to handle a large diversity of symbols in various experimental contexts (Delaye and Anquetil, 2013). In contrast to (Willems and Niels, 2008), Delaye and Anquetil only consider the simplest features from each feature category (geometrical, temporal, etc.), to maintain a feature space of limited dimension (Delaye and Anquetil, 2013). They evaluate their 49 features using a standard SVM and a simple 1-Nearest-Neighbor classifier on eight data sets with considerable diversity of content (digits, characters, symbols, geometrical shapes and gestures). Results show that using off-the-shelf statistical classifiers, the HBF49 representation performs comparably or better than state-of-the-art results reported on these hand-drawn objects (more than 90% accuracy using SVMs).

Another set of 14 features described by Sonntag et al. (2014), presented as part of a pen-based interactive decision support system for radiologists, is included in the selection. A common practice in hospitals is that a radiologist's dictated or written patient report is transcribed by hospital staff and sent back to the radiologist for approval, which takes a lot of time and lacks direct digitization of pen input. Sonntag et al. present a system, which allows doctors to use a digital pen to fill out the paper-based structured reporting form, with direct digitization of pen input in real-time. The input is not limited to numbers and text, but can also include hand-drawn sketches, free text annotations and correction gestures. Instead of forcing the user to switch manually between writing, drawing, and gesture mode, a mode-detection system is deployed to predict the user's intention based on the sketch input. To classify the input, a number of features are calculated, including compactness, eccentricity, closure and others. These features are used in a multi-classification and voting system to detect the classes of handwritten information, shape drawings, or pen gestures (Sonntag et al., 2014). According to Sonntag et al., the system reaches a recognition rate of nearly 98%. Their work shows how digital pen features can be used transparently in pen-based intelligent user interfaces to improve interactivity and reduce cognitive load for the user.

So far 165 digital pen features from the four aforementioned feature sets (Rubine, 1991; Willems and Niels, 2008; Delaye and Anquetil, 2013; Sonntag et al., 2014) are included in the selection. In addition, 11 features are considered, collected from literature focused on the evaluation of cognitive assessments (Werner et al., 2006; Cohen et al., 2014; Davis et al., 2015). These features include the number of strokes, sketching time, stroke distance, duration, average pressure, average velocity, the variation of velocity, number of pauses, average pause duration, the ratio between sketching and pausing, and average lift duration. A comprehensible summary of these publications is given in section 2.3 (Analyzing Cognitive Tests Using Digital Pen Features). By directly analyzing the characteristics of pen input and sketching behavior, a causal link between sketch characteristics and cognitive test performance is created, without the need to analyze the sketch content itself. This way cognitive behavior is classified transparently during user interaction and independent of the task at hand, thereby enabling additional opportunities in interactive systems, such as evaluation, feedback and content adaptation of pen-based interfaces in real-time.

In summary, we consider a total of 165 (+11) digital pen features, which are computable in real-time, and, among others, cover geometrical, spatial, temporal, and pressure properties of sketches. The complete list of features is provided in the Appendix section.

### 2.2. Cognitive Assessments

Neurocognitive testing is a noninvasive method for measuring brain function by evaluating specific cognitive abilities, including memory, fine motor control, reasoning and recognition. Despite recent technological advances, the majority of cognitive assessments used in practice is still conducted using pen and paper with manual scoring by the physician afterwards. This includes verbal interview like tests, in which the physician takes notes of the patient's answers, as well as tests in which the patient is asked to write or sketch. In this work, a selection of paper-pencil cognitive assessments is considered based on feedback from domain experts and a recent market analysis of existing, widely used, cognitive assessments conducted by Niemann et al. (2018). These assessments were successfully digitalized during the Interakt project (Interactive Cognitive Assessment Tool) (Sonntag, 2017), and are summarized in Table 1, namely Age-Concentration (AKT) (Gatterer et al., 1989), Clock Drawing Test (CDT) (Freedman et al., 1994), CERAD Neuropsychological Battery (Morris et al., 1988), Dementia Detection (DemTect) (Kalbe et al., 2004), Mini-Mental State Examination (MMSE) (Folstein et al., 1975), Montreal Cognitive Assessment (MoCA) (Nasreddine et al., 2005), Rey-Osterrieth Complex Figure (ROCF) (Duley et al., 1993), and Trail Making Test (TMT) (Reitan, 1986). This selection of tests accounts for a variety of patient populations and test contexts. In this thesis, the focus is on the CDT, the TMT and the ROCF, as they have the highest ratio of pen input relevant to the scoring result. The AKT is discarded, because it only consists of the rather simple task of crossing out figures, and preliminary testing results show that the samples have too few strokes for analysis.

**Table 1:** Comparison of widely used cognitive assessments including the percentage of tasks with pen input that contribute to the total score.

**Assessment**	**Execution time**	**Pen input**	**Sketch shapes**
AKT (Gatterer et al., 1989)	15 min	100%	Cross-out
CDT (Freedman et al., 1994)	2–5 min	100%	Clock, digits, lines
CERAD (Morris et al., 1988)	30–45 min	20%	Pentagons, rectangles, circle, ...
DemTect (Kalbe et al., 2004)	6–8 min	20%	Numbers, words
MMSE (Folstein et al., 1975)	5–10 min	9%	Pentagons
MoCA (Nasreddine et al., 2005)	10 min	17%	Clock, digits, lines
ROCF (Canham et al., 2000)	15 min	100%	Circles, rectangles, triangles, ...
TMT (Reitan, 1986)	3-5 min	100%	Lines

*Execution time does not include the time needed by the physician to score the test (several minutes, depending on test)*.

**Figure 1:**
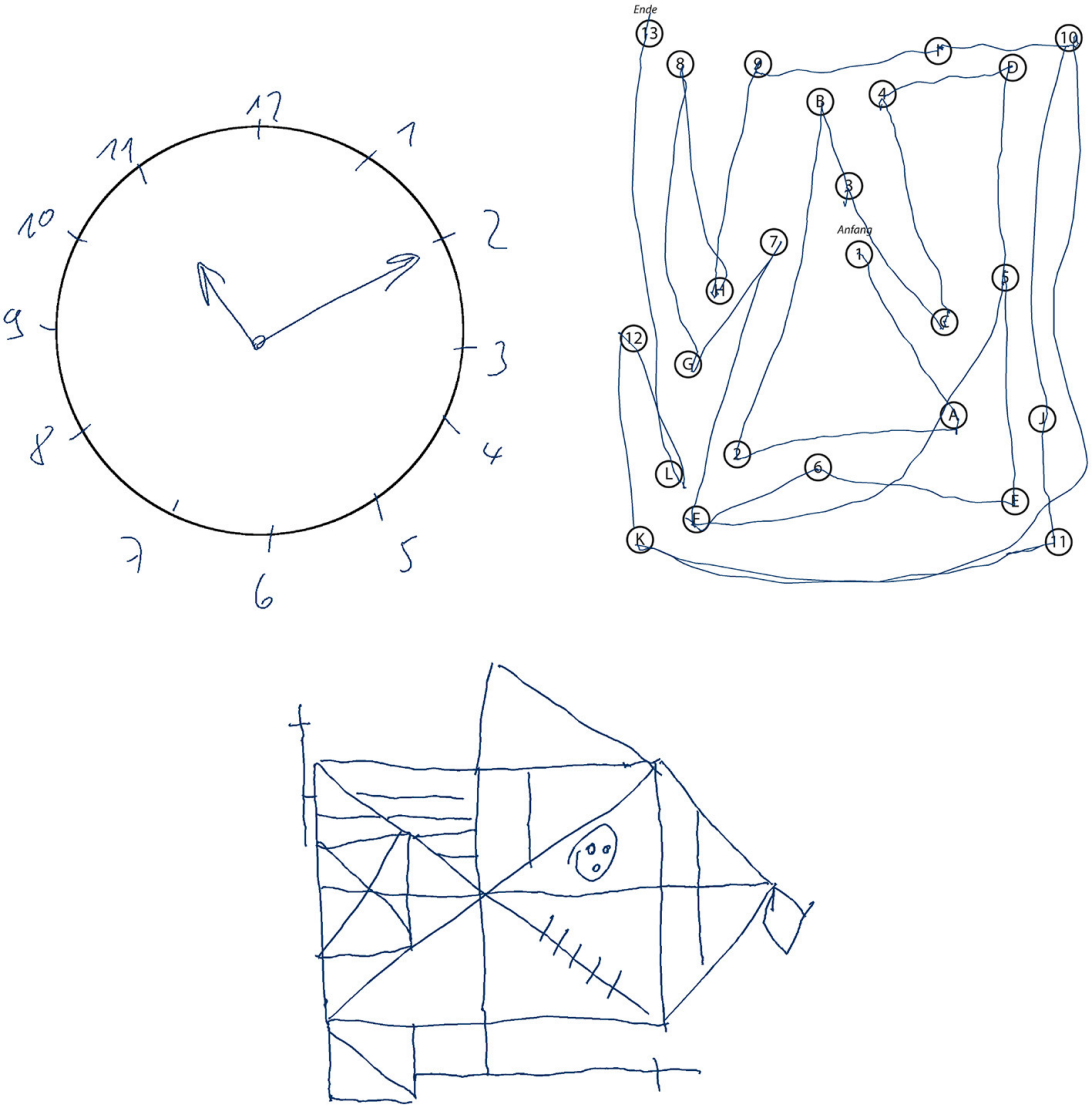
Samples of cognitive assessments recorded during the Interakt project: Clock Drawing Test (upper left), Trail Making Test B (upper right), and Rey-Osterrieth Complex Figure Test (bottom).

For more than 50 years, the CDT is used as an assessment tool for cognitive impairment. It is a simple paper and pencil test in which the participant is asked to draw a clock face and indicate a certain time (see upper left of Figure 1). The task is primarily designed to test the visuospatial ability and is often used in geriatrics to screen for signs of dementia, such as Alzheimer's disease, or other neurological conditions, including Parkinson's disease, traumatic brain injury, and stroke recovery. Usually a trained professional observes the clock drawing task and scores the final sketch based on a scoring scheme, which takes up to a few minutes. Automatically scoring the CDT has several benefits: Firstly, it significantly reduces the time caregivers have to invest into administering the CDT; and secondly, it is likely to produce more objective scores and potentially enables a more detailed analysis (Sonntag, 2017). Not all scoring schemes are equally well suited for automation, since most of them have been designed to be quick and easy to be interpreted by human testers. The 20-point Clock Drawing Interpretation Scale (CDIS) by Mendez et al. (1992) is selected in this thesis, because it contains clear test parameters that can be modeled mathematically and computationally. In addition, the manual scoring procedure of CDIS is very time-consuming and would highly benefit from automatic computation. The CDIS contains items such as “All numbers 1–12 are present,” which are to be rated 0 if not fulfilled and 1 if satisfied. All 20 individual scores are then added up and the final score indicates the severity of cognitive impairment. For example, a score of 18 or less is likely to indicate Alzheimer's and similar forms of dementia.

Frequently used in neuropsychological testing, the Trail Making Test (TMT) (Reitan, 1986) is a standardized paper and pencil test in which the participant is asked to connect numbered nodes similar to a child's connect-the-dots puzzle (see upper right of Figure 1). In its original form, it was part of the Army Individual Test Battery (1944) and was later subsequently incorporated into several cognitive test batteries (Tombaugh, 2004). The TMT is widely available and can be easily administered in practice (Dahmen et al., 2017). In addition, it can be used to assess a variety of neurological disorders (Salthouse, 2011). Here, the use case of geriatrics is considered, where TMT versions A and B are used to screen for signs of dementia. Each of the two parts (A and B) consists of 25 encircled items on an A4 sheet of paper and subjects are asked to draw a line through them in the correct order as quickly as possible without lifting the pen. TMT-A involves number sequencing (1 to 15), whereas TMT-B includes set-shifting: it requires the subject to alternate between numerical and alphabetic sequences (1A2B3...). The main performance indicator used in clinical practice is the total completion time, which is manually measured by the physician using a stopwatch (Lezak et al., 2004; Bowie and Harvey, 2006). The total scoring of the cognitive assessment is then calculated by comparing the completion time to age-specific normative values (Lezak et al., 2004; Dahmen et al., 2017).

**Figure 2:**
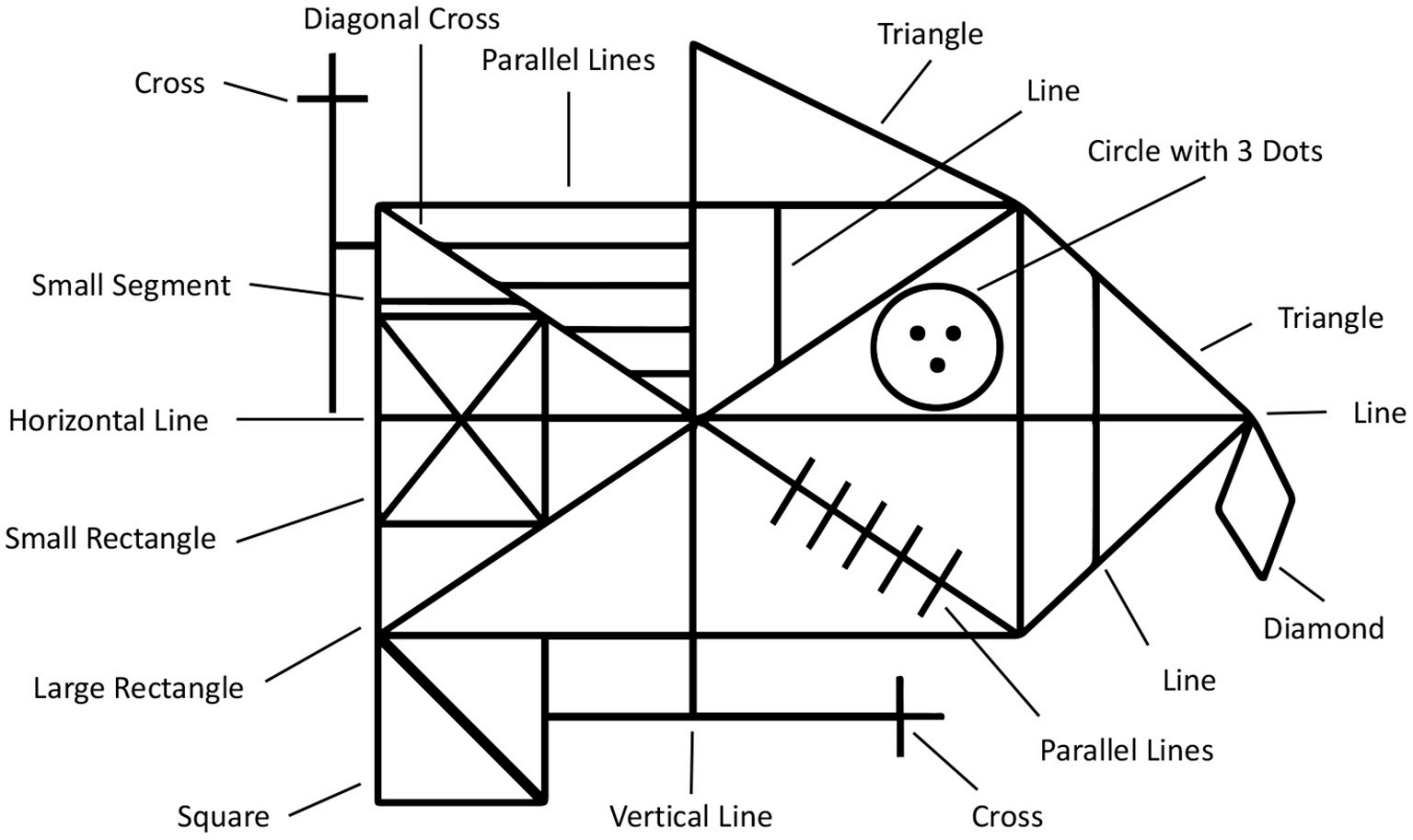
The Rey-osterrieth complex figure (ROCF) is composed of 18 sub-figures.

An even more complex example of a neuropsychological assessment, that is rated entirely based on pen input, is the ROCF (Duley et al., 1993; Canham et al., 2000). A printed Rey-Osterrieth figure template is presented to the subject, who is then asked to copy it onto a blank piece of paper (see bottom of Figure 1). Then the physician hides the template and the subject is asked to immediately recall the figure from memory and sketch it onto another blank piece of paper. This is repeated once again after approximately 30 min (delayed recall). Afterwards the physician scores each sketch based on the visual appearance of the 18 sub-figures of the ROCF (see Figure 2). Each sub-figure is rated between 0 (non existent) and 2 (drawn and placed correctly), and the individual scores are summed up to produce the final score. In contrast to the CDT, the TMT and ROCF have only one scoring scheme that is commonly applied in practice according to medical guidelines.

### 2.3. Analyzing Cognitive Tests Using Digital Pen Features

Cognitive assessments have been the subject of recent debate because there are limitations when they are conducted using pen and paper. For example, the collected material is monomodal (written form) and there is no direct digitalization for further and automatic processing. Additionally, the results can be biased depending on the physician's level of expertise. To mitigate these shortcomings, digitizing and analyzing paper-and-pencil assessments has been introduced recently (Sonntag, 2018). Using a digital pen to record neuropsychological tests allows for the analysis of additional parameters that cannot be considered otherwise. In Heimann-Steinert et al. (2020) we show that replacing a standard ballpoint pen with a digital pen has no influence on neurocognitive test results. Werner et al. (2006) perform an experiment in which they record common cognitive assessment tasks using a digitizer tablet to compare the handwriting behavior of healthy controls to patients suffering from mild cognitive impairment (MCI) and Alzheimer's disease. Their feature set includes on paper time, in air time, the ratio between in air and on paper time, on paper length, velocity and pressure. They report significant differences between the groups in almost all measures (Werner et al., 2006), for example, temporal measures are higher and pressure is lower in the cognitively impaired groups. Using ANOVA, Werner et al. classify 69–72% of the participants correctly, although the classification for the MCI group is reported to be relatively poor. Findings by Schrter et al. (2003) support the statement that it is possible to distinguish between different forms of cognitive impairment and healthy subjects by analyzing handwriting movements. They find that both patients with MCI and patients with probable Alzheimer's exhibit loss of fine motor performance and that the movements of Alzheimer patients are significantly less regular than those of healthy controls. In both publications the classification is made entirely without content analysis, solely based on the handwriting behavior of subjects.

Similar findings are reported by Davis et al. (2015), who use an off-the-shelf digitizing ballpoint pen to analyze the cognitive abilities of patients with dementia, Alzheimer's and Parkinson's disease, by deploying a digital version of the CDT. A set of digital pen features, including temporal aspects, such as speed and pauses, is used as input for a collection of ML algorithms, including SVMs, random forests, boosted decision trees, and others. They report a top accuracy of 82% using SVMs to classify dementia, but it is not clear which features exactly are used. However, Davis et al. report that time-dependent variables prove to be important for detecting cognitive change. They can reveal when individuals are working harder, even though they are producing normal-appearing outputs, e.g., the total time to draw the clock differentiates those with amnestic mild cognitive impairment (aMCI) and Alzheimer's disease from healthy controls (Davis et al., 2015). In Cohen et al. (2014) the same authors elaborate on these findings by comparing the ratio of drawing vs. non-drawing time for older adults with depression during the CDT. Their evaluation reveals a significant effect of age on drawing times during challenging tasks, while there is no significant effect during simpler tasks like copying figures. This could prove highly relevant for interactive pen-based systems, in which content and task difficulty need to be adjusted in real-time, depending on the cognitive load of users. Oviatt et al. report similar findings in the educational domain, where they use digital pen features to predict task expertise and user performance in mathematics (Oviatt and Cohen, 2014; Zhou et al., 2014; Oviatt et al., 2018). Using SVMs, Random Forests, and Naive Bayes classifiers, they achieve up to 92% prediction accuracy with features, such as average number of pen strokes, total writing time, stroke distance, duration, pressure, and speed. Similar techniques have been used to diagnose other cognitive impairments, such as Parkinson's disease (Drotár et al., 2014; Kuosmanen et al., 2020), or to predict task difficulty and user performance of cognitive tests (Barz et al., 2020). This shows that the ML-based digital pen features approach considered in this work can be applied to a variety of application domains and use cases.

Digital pen features are also useful to perform a traditional content analysis of cognitive assessments. Automatic scoring systems mimic the scoring procedure performed by physicians, which is regulated by medical guidelines and usually produces a numeric score. In Prange and Sonntag (2019) we show how to model cognitive status through automatic scoring of a digital version of the CDT. We implement the Mendez scoring scheme and create a hierarchy of error categories that model the test characteristics of the CDT, based on a set of impaired clock examples provided by a geriatrics clinic. Using a digital pen we record 120 clock samples for evaluating the automatic scoring system, with a total of 2,400 samples distributed over the 20 scoring classes of the Mendez scoring scheme. Samples are scored automatically using a handwriting and gesture recognition framework based on digital pen features and results show that we provide a clinically relevant cognitive model for each subject. In addition, we heavily reduce the time spent on manual scoring. A similar automated analysis of the CDT based on ML is presented by Souillard-Mandar et al. (2016). They design and compute a large collection of content related features and explore the performance of classifiers built using a number of different subsets of these features and a variety of ML techniques. The authors use traditional ML methods to build prediction models that achieve high accuracy in detecting cognitive impairment. However, their major drawback is, that the deployed feature set is not reproducible, nor is there a data set available for repeating the experiments.

Binaco et al. (2020) present an approach that uses the digital CDT (Davis et al., 2010; Penney et al., 2010), a special version of the CDT which includes the same instructions and administration procedures as the original, and where they collect pen data using the Anoto digital pen. The pen is very similar to the Neo smartpen N2 we are using in our proposed system. Both pens are capable of recording timestamped X/Y stroke data including applied pressure. Binaco et al. perform feature selection on a set of more than 300 features to train a neural network that differentiates between mild cognitive impairment sub-types and Alzheimer's disease. However, the authors do not explicitly state which features they use and how they are extracted, thus making their approach hard to reproduce, i.e., there are several approaches on how to compute and count individual features. For example, a feature like the length of a stroke could be computed for every of the 15 sub-parts of the clock (12 digits, 2 hands, and 1 circle), resulting in 15 features in total. In contrast, in our approach we do not use specific test-dependent domain knowledge, but calculate features like length across the entire figure, resulting in one feature. This allows us to apply the same features and methods on different cognitive tests. In a subsequent publication (Davoudi et al., 2021) the authors use 37 of the dCDT features to classify non-dementia and Alzheimer's disease/vascular dementia patients. Their list of features includes general as well as CDT-specific features, including the total number of strokes, completion time, average pressure, but also clock hand lengths, average digit height, clockface area or “any digit over 12.” Both related works provide excellent examples of feature sets that are hand-crafted and fine-tuned toward a specific cognitive test, and which show promising ML classification results for the analysis of digital pen data.

Another very popular assessment is the Mini-Mental State Examination (MMSE) introduced in Folstein et al. (1975). The MMSE is a 30-point questionnaire, which is administered by a trained professional, who leads the subject through the questionnaire and sketching tasks whilst taking notes. The CDT is often administered together with the MMSE, since both assess different, complementing cognitive abilities (Palsetia et al., 2018). In Prange et al. (2019) we present a multimodal system for the automatic execution and evaluation of the MMSE, that uses speech analysis in combination with handwriting and gesture recognition based on digital pen features. Taking into account multiple sensor inputs is the next step to improve neurocognitive testing by taking advantage of multimodal, multi-sensor interfaces (Oviatt et al., 2017). In Niemann et al. (2018) we describe the architecture of a multimodal multi-sensor assessment framework for cognitive impairment that combines digital pen strokes, pen sensors, automatic handwriting and gesture recognition and electrodermal activity (EDA). We use EDA for potential inclusion of open sympathetic arousal, stress and affect (e.g., emotion detection through autonomic nervous system signals). To include biosensors (e.g., EDA) into future digital pen-based interfaces is very interesting because it is a process-tracing method (unobtrusive and continuous measure) for neural activity and can reflect psychological processes. The digital pen-based environment provides a sensor fusion context for its interpretation. In the future, large scale community screening programs can arise from resulting multimodal data collections to identify multimodal profiles of impairment across different cognitive, psychiatric and functional domains/abilities. It will also help to guide differential diagnosis and further neurocognitive assessment, especially because multimodal digital assessments are unbiased to a large degree.

Clinically relevant examples of digitalized pen-based cognitive assessments also include the ROCF (Duley et al., 1993; Canham et al., 2000), which can be used for various purposes, such as diagnosing the periphery (Coates et al., 2017). The authors show that accurate reproduction of spatial features predictably declined as the target was presented further in the visual periphery. Such analysis of the periphery in the context of the ROCF drawing tasks has shown relevant for a variety of medical conditions, including age-related macular degeneration and apoplectic stroke (Coates et al., 2017). Wang et al. (2017) report a significant correlation between ROCF measures, tremor and bradykinesia (impaired and slow body movement), such as is often present in patients suffering from cognitive impairment (i.e., Parkinson's disease). Similarly, Salthouse (2011) shows that the TMT can be used to assess a variety of neurological disorders and it can even be adopted for diagnosing neurological disorders in children (Reitan, 1971). His findings are backed by Barz et al. (2020), who use digital pen features to predict task difficulty and user performance of the TMT. Their automated evaluation uses a subset of the features considered in this thesis and shows that a correlation-based feature selection can be beneficial for ML-based model training in certain scenarios. Similar results were reported by Dahmen et al. (2017), who investigated the utility of features in analyzing a digital version of the TMT. Their considered features include, among others, the average pause duration, number of lifts and average pressure.

Pereira et al. (2016) present a deep learning approach where they transform the raw digital pen time-series sensor data (i.e., tilt, acceleration and pressure) into gray-scale images. The resulting images are used to train a convolutional neural network (CNN) for the classification of Parkinson's disease. Instead of creating artificial images from the sensor data, several deep learning approaches exist that aim to classify cognitive performance based on the scans or rendering of the actual sketches. Moetesum et al. (2020) use CNNs for the classification and modeling of deformations in cognitive assessments, including the CDT and ROCF. In similar approaches Amini et al. (2021) and Youn et al. (2021) use CNNs to predict cognitive impairment from the CDT and ROCF. Chen et al. (2021) compare different CNN architectures like VGG16, ResNet-152 and DenseNet-121 for automatic dementia screening and scoring of the CDT. A combination of image-based analysis and sensor data is presented by Park and Lee (2021), who utilize CNN and U-Net architectures for classification and segmentation of hand-drawn CDT sketches. One of the drawbacks of these approaches is that most deep learning models are inherently black-box, making it difficult for clinicians to judge the classification results, whereas models that utilize digital pen features provide more transparent results.

To summarize, utilizing digital pen features, in ML-based automated cognitive assessment systems, opens up an opportunity to analyze handwriting and sketching behavior, that could not be considered otherwise. Related work shows that these features can be used for both, sketch content analysis and the unobtrusive, transparent modeling of cognitive behavior from raw digital pen input.

## 3. Data Collection Method

In the medical domain, pen-based neurocognitive testing is used to assess a wide range of cognitive impairment, including dementia, Parkinson's disease or traumatic brain injury. Usually a trained professional observes the test and scores the final sketch based on a scoring scheme, which takes up to a few minutes. An automatic scoring system has several benefits. First, it significantly reduces the time caregivers have to invest in administering the assessment. Second, it is likely to produce more objective scores and potentially enables a more detailed analysis. Therefore, this section presents a cognitive assessment tool that is used to record a sketch data set of cognitive assessments with the goal to automatically analyze test performance based on digital pen features.

### 3.1. Interactive Cognitive Assessment Tool Architecture

**Figure 3:**
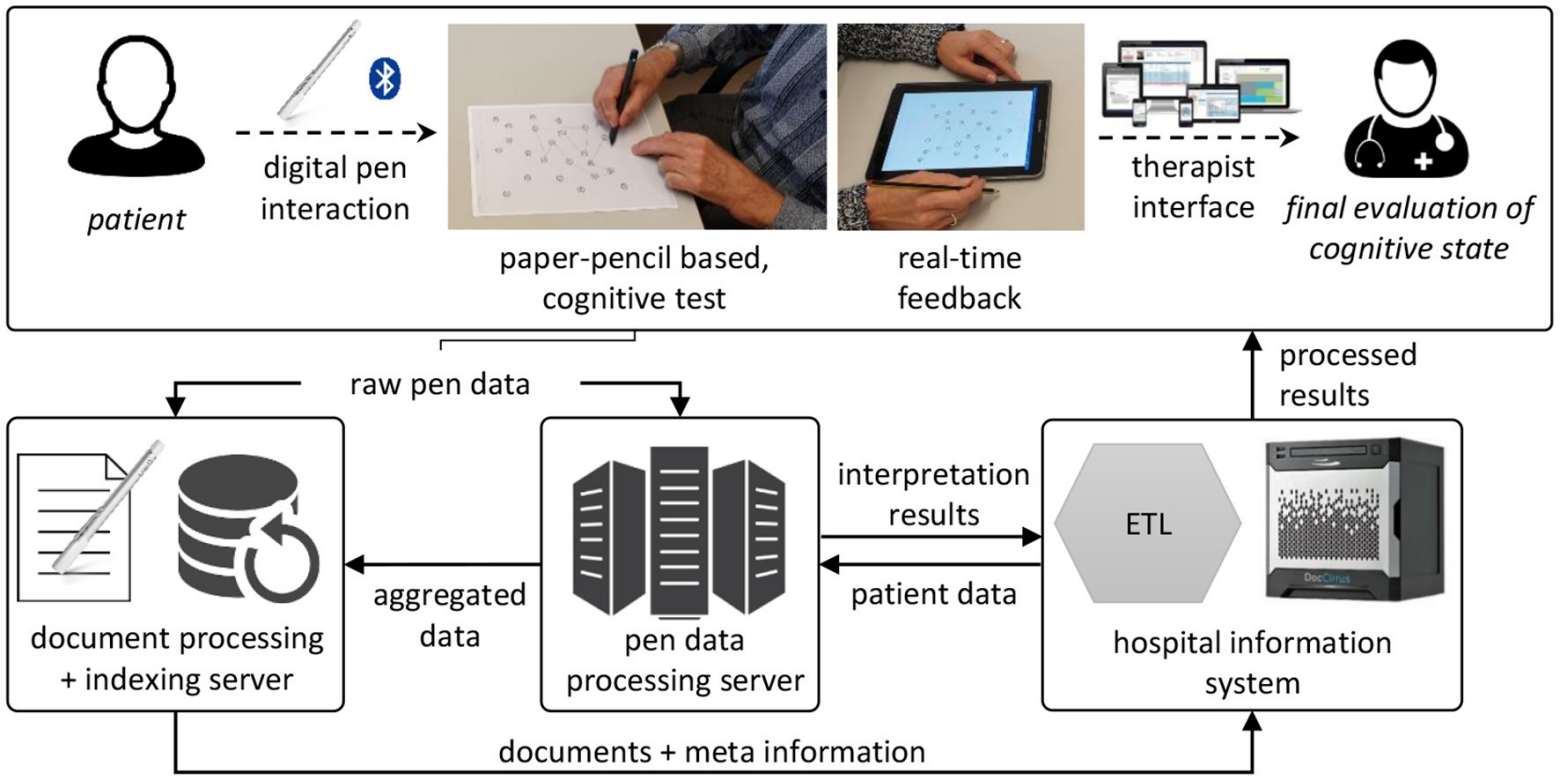
Architecture of the interactive cognitive assessment tool. Data is recorded using a digital pen and streamed to a backend service, where it is analyzed before being loaded into the hospital information system and presented to the physician. ETL, extract, transform, load; a general concept of automatic data processing.

Figure 3 provides a general overview of the automatic cognitive assessment tool for the CDT, TMT and ROCF. It is fully implemented and currently being deployed in a geriatrics daycare clinic in Berlin. As prerequisite, digital versions of the original assessment forms are created by scanning and then overlaying the original with reference points and bounding boxes, e.g., for each of the encircled nodes of the TMT. The Neo smartpen N2^1^ and its Euclidean 2D coordinate space are used to record the sketch samples. With a built-in infrared camera, the N2 digital pen tracks its position on the piece of paper, by recognizing a micro-dot pattern, that is printed on the paper forms. The pattern is almost invisible to the human eye and can be printed by standard laser printers, such as they are present in hospitals, clinics and medical offices. This approach does not require special software, the forms are printed directly from a PDF file. A physician connects the digital pen via Bluetooth to a mobile application, which can be run off any common Android smartphone or tablet and is connected to the hospital information system. Recorded handwriting data is streamed in real-time to the tablet, where the digital pen strokes are directly rendered on the screen, giving instant feedback to the physician. This setup has the potential benefit that physician and subject do not need to sit close together, e.g., in telemedicine applications or due to Covid-19 distancing regulations.

**Figure 4:**
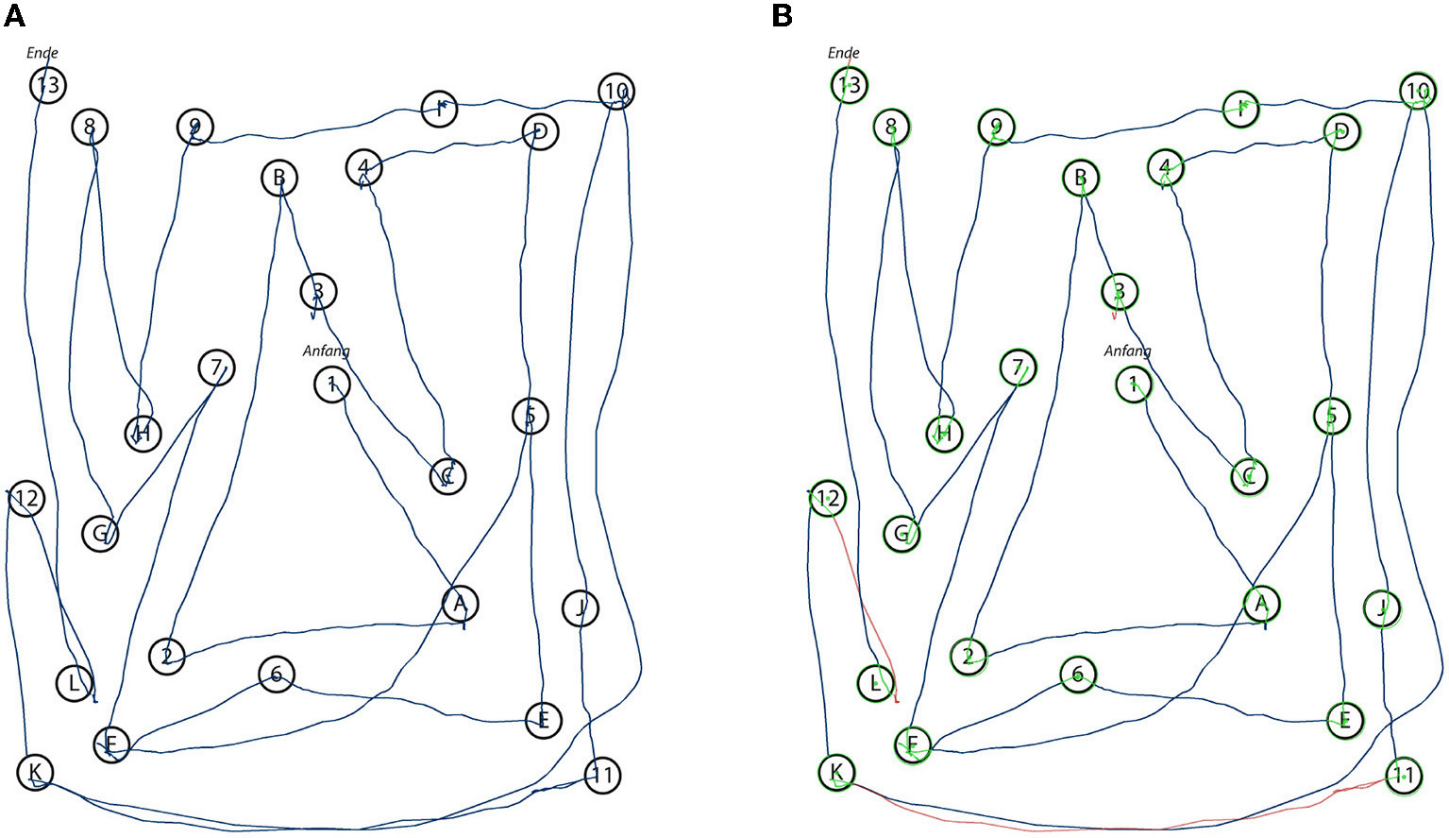
Renderings of the original input **(A)** as recorded by the digital pen during the data collection with a subject performing the TMT-B. The automated system analyzes the input and annotates the stroke data with the information relevant for the algorithm's final scoring decision **(B)**. Green dots indicate the center points of the circles and green stroke parts indicate that a circle has been successfully hit by a stroke, while red strokes could not be identified as connecting two circles.

**Figure 5:**
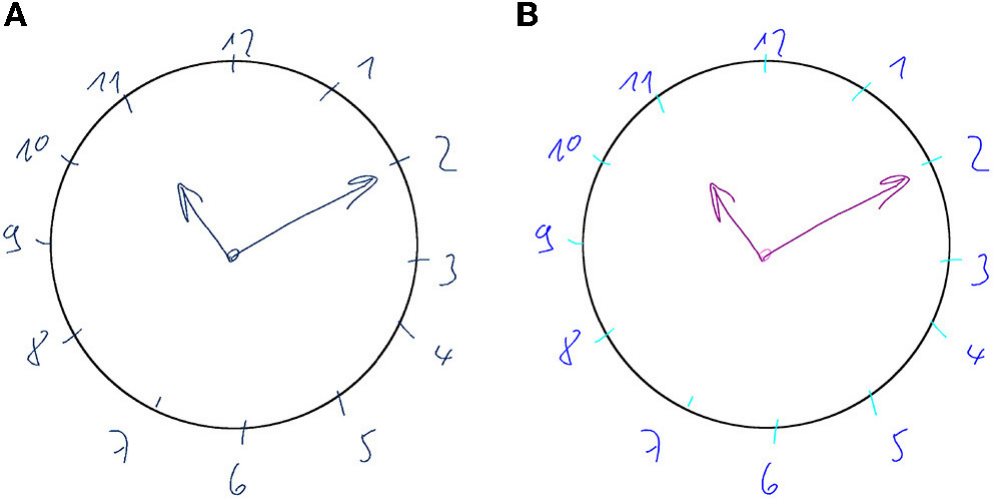
Renderings of the original input **(A)** as recorded by the digital pen during the data collection with a subject performing the CDT. The automated system analyzes the input and segments the strokes into classes **(B)** (digits, helper lines, center point, hands etc.). Using this segmentation the system automatically performs a scoring based on the 20-point CDT scoring scheme by Mendez et al. (1992).

**Figure 6:**
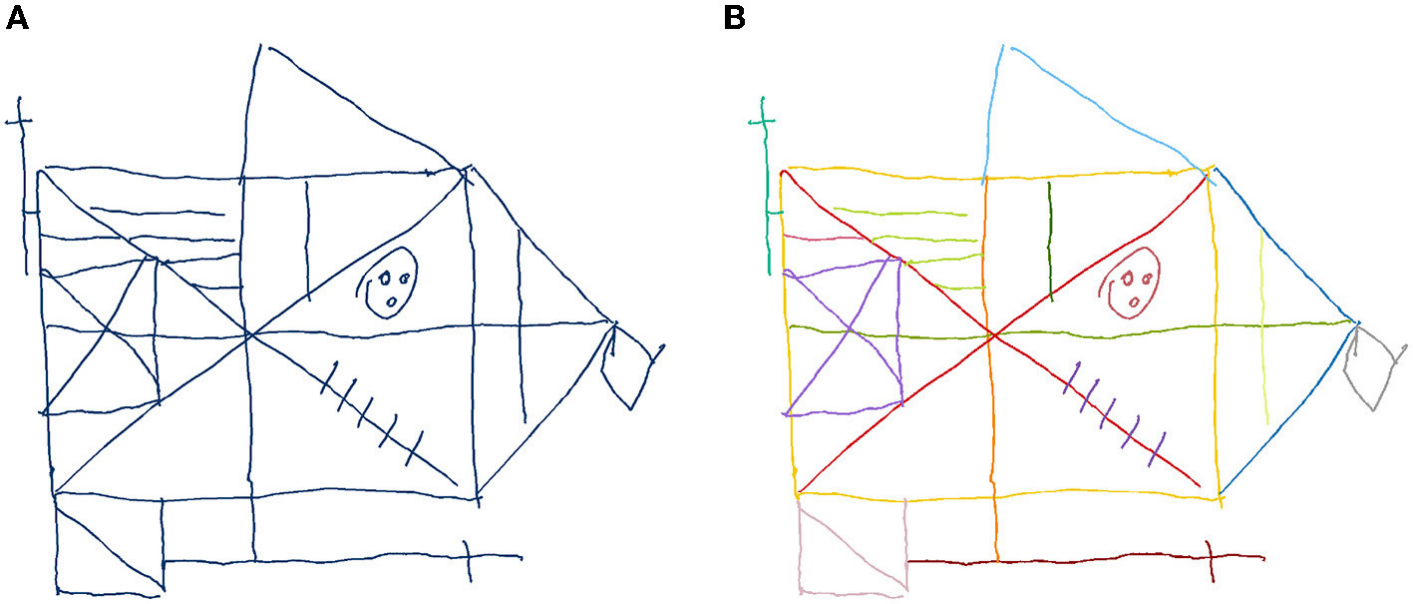
Renderings of the original input **(A)** as recorded by the digital pen during the data collection with a subject performing the ROCF. The automated system segments the strokes into the 18 ROCF sub-figures, which are represented by different colors **(B)**. Based on this segmentation the algorithm scores the individual sub-figures, from 0 = “not present,” to 2 = “correct” (Duley et al., 1993).

The raw pen data is exported to a backend service, which creates a digital copy of the document for indexing and documentation purposes. The pen data processing server analyzes the pen strokes as a series of time-stamped two-dimensional coordinates. In case of the TMT, it matches the input to the reference template of the original TMT, thereby reproducing the path taken by the subject (see Figure 4B). During this process, the algorithm produces transparent explanations for encountered errors and extracts missing connections. The completion time, which is the major scoring criterion for the TMT, is calculated for each sample by subtracting the timestamp of the first recorded pen stroke point from the last recorded timestamp. For the CDT the strokes are segmented into semantic classes, such as digits, helper lines, center point, hands etc. (see Figure 5B). Based on this segmentation the system generates scores following the 20-point CDIS scoring scheme by Mendez et al. (1992). Similarly, the ROCF sketches are segmented into the 18 sub-figures of the ROCF (see Figure 6B) and scored according to the official scoring scheme, whose score is based on the individual scores of the sub-figures (Duley et al., 1993; Canham et al., 2000).

For each subject, the results of the scorings are summarized in a structured PDF document, which includes the completion time, the original rendering of the recorded data, the annotated version of the analyzed data and detailed information about the scoring results. Providing a rendering of the unaltered sample serves (Figures 4A, 5A, 6A) both documentation and transparency requirements: physicians need to be able to examine the original and to have a clear understanding of what was generated by the system. To further increase the transparency of the automatically generated scoring, the physician is presented with a representation of what the algorithm detected, e.g., for the TMT the reference points are indicated in green and strokes are colored in red when recognized as not connecting any two nodes (see Figure 4B). For the CDT and ROCF, the segmented sketches are included (see Figures 5B, 6B). This kind of transparency is highly relevant to the physicians, as they need to be able to judge whether the automatic scoring, they are relying on, is accurate and can be trusted. Similarly, the focus of this thesis is on the analysis of digital pen features to predict test performance using ML, which can be used to produce models that can be explained by the features themselves.

### 3.2. CDT, TMT, and ROCF Data Collection Method

The data set is collected during a repeated measures experiment with 40 participants, who are recruited from the geriatrics daycare clinic of a large German hospital in Berlin. Inclusion criteria for participants are a minimum age of 65 and the signed informed consent. Exclusion criteria are severe cognitive disorders, mental diseases, severe auditive, visual, linguistic, sensory or motor limitations, chronic pain or a legal representative. A total of 40 older adults are included (age *M* = 74.4 ± 4.1 years, range: 67–85 years) in the experiment, of which half are female. The majority of participants is well-educated (57.5% high-level education) and right-handed (95.0%). A total of 25 participants (62.5%) rate their health as rather or very good, whereas 22 participants (55.0%) report to suffer from a chronic disease such as diabetes (*N* = 8) or hypertension (*N* = 5). When asked afterwards, whether the type of pen influences their test performance, almost all participants (95.0%) answer the question in the negative.

**Figure 7:**
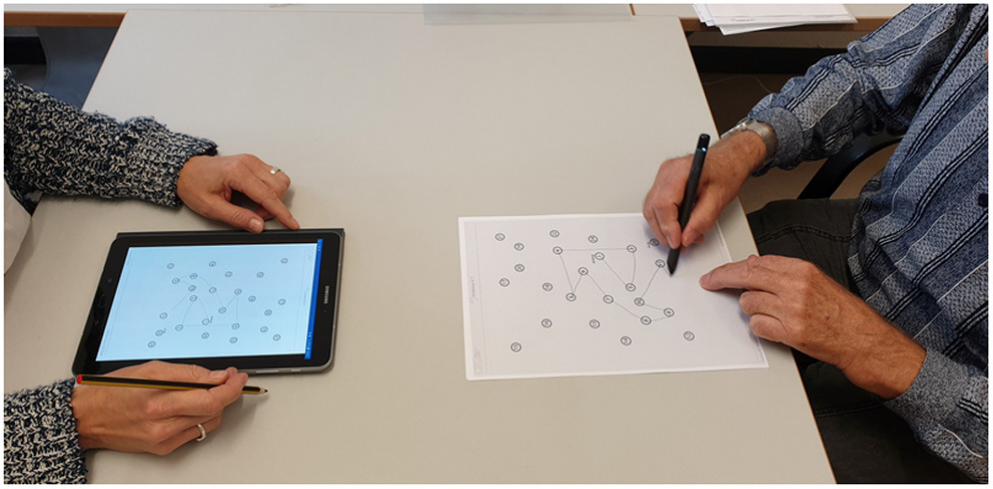
The data collection setup in the geriatrics daycare clinic. The subject (right) performs the cognitive test (here TMT) using a digital pen, which is connected via Bluetooth. The result can be monitored in real-time on the tester's tablet (left).

The experiment has two conditions, paper-pencil and digital pen on paper. The versions of the cognitive assessments are the same in both conditions, just the type of pen differs. For each condition, participants perform a total of 6 tasks [CDT, TMT (A and B), and ROCF (copy, immediate recall and delayed recall)]. To avoid order effects, the execution order is balanced: half of the participants start with the paper-pencil version first and the other half with the digital pen. Participants sit in a distraction-free room at a table with the pen and the test in front of them (see Figure 7). The physician sits on the opposite side of the table and holds a tablet, to which the digital pen is connected and on which the participant's input can be tracked in real-time. The feedback is only visible to the experimenter. The tablet records the data, which is streamed via Bluetooth by the digital pen, for later analysis. An important factor for successful deployment is the seamless integration of digital medical applications into everyday hospital processes. The system supports this requirement in several ways. First, the tablet application and digital pen are highly mobile and can directly be taken into the examination room, or to the patient's bedside, if necessary. Second, the application has access to a list of patients from which the physician can choose and initiate an assessment, thereby directly linking the digital test to the correct patient file. If required for the analysis, the backend service can access patient data, such as demographic data (e.g., age) or previous assessments for a comparison of cognitive performance at different points in time. Third, all structured reports, generated during analysis, are sent to the hospital information system, these include the generated PDF report, the same information in a structured file format for direct use in database systems and a replay video of the digital pen recording. Lastly, the physician can access and query all information in a web-based user interface.

Participants are given the original test instructions for each task (Reitan, 1992; Duley et al., 1993; Freedman et al., 1994). For each condition they are asked to first perform the ROCF (copy and immediate recall), then the TMT-A and TMT-B (the order is given by the TMT's test design), followed by the CDT and finishing with the ROCF (delayed recall). By letting participants perform the other tests in between the ROCF tasks, the physician makes optimal use of the time, as the test design requires a pause between recall and delayed recall. The instructions for the tests are given by a trained physician. In both modes (normal and digital pen) the TMT task execution time is measured manually by the rater using a stopwatch. There is a wash-out phase between the modes of approximately 30 min. During this phase, participants complete a self-developed questionnaire to collect socio-demographic data and a questionnaire to record the technology commitment (Neyer et al., 2012).

The collected data set consists of a total of 240 sketches (40x CDT, 80x TMT and 120x ROCF). Four samples of the ROCF are discarded, in which participants did not sketch anything and for which therefore it is not possible to calculate any features. After removing noise (e.g., notes and scribble) from the sketches, the stroke coordinates are translated to the origin of the Cartesian coordinate system (*min*(*x*) = 0, *min*(*y*) = 0). No scaling or pre-processing is applied to the sketches to avoid influencing participants' sketching characteristics. Each sketch is analyzed manually by a physician in accordance with the medical guidelines. For the TMT the score is based on the measured execution time in seconds (Reitan, 1986), while the 20-point Mendez CDIS scheme (Mendez et al., 1992) is applied to the CDT and the original 36-point scoring system for the ROCF (Duley et al., 1993). In the considered use case of geriatrics, the scorings are used as an indicator to assess whether or not to schedule further cognitive testing procedures. Therefore, each sketch is classified into one of two classes, healthy and suspicious, based on the scoring results. Dependent on the execution time the TMT-A and TMT-B samples are classified as suspicious if they take over a minute or over 2 min respectively. Samples of the CDT are labeled as suspicious if the score is 18 or less, whereas ROCF samples are considered suspicious with scores of 30 or less. In total 152 samples are labeled as healthy and 84 as suspicious.

## 4. Evaluation

Our evaluation is concerned with the ML-based classification of cognitive test performance. Cognitive assessments are commonly used in medicine to either diagnose disease (e.g., dementia, Parkinson's, etc.) or to screen for signs of cognitive impairment, e.g., as part of large-scale community screening programs. In both use cases, the underlying task is to categorize subjects either as healthy or as suspicious, the latter of which would indicate to conduct additional testing or medical interventions. Traditional approaches for digitalizing cognitive assessments are described in previous sections. These methods are proven to be successful for a wide variety of cognitive assessments, but they are also highly dependent on the specific test and often analysis components include task-related semantic knowledge that needs to be modeled manually for each type of test. The next big step in analyzing digital cognitive assessments is to predict cognitive performance independently of the test content, by looking only at the writing and sketching behavior of users. This approach allows us to generalize the classification of cognitive performance independently of the considered task and opens up new approaches for transparent behavior analysis in pen-based intelligent user interfaces. Users do not longer have to perform specific tests, instead, their handwriting and sketch input can be analyzed transparently during common pen-based tasks like sketching, taking notes or writing messages. In the future, such a prediction of cognitive performance can be used in interactive pen-based systems to adapt task difficulty and content in real-time, depending on the cognitive state of the user. This is why the last experiment aims at predicting cognitive performance by only considering the syntactic digital pen features, which are applicable independent of the task, without performing further content analysis.

### 4.1. Methodology

Our cognitive assessments data set contains a total of 236 sketch samples of the CDT, TMT and ROCF. The samples were manually scored and annotated by experts, who labeled each sample as either healthy or suspicious. Based on the task design all previous experiments considered only sketch-based digital pen features, meaning that the entire sketch was used as input for each of the feature functions to produce the feature vector. However, many features, especially all of Rubine's features (Rubine, 1991) can be applied on a stroke-level as well. Taking into account stroke-level features as well serves a double purpose. Firstly, many gestures and symbols consist of only one stroke anyways and the research question arises, whether or not one can predict cognitive performance from the sketching behavior of as little data as single strokes, or if entire sketches are required. Secondly, due to unforeseen circumstances in regard to the global pandemic, the data collection had to be cut short, resulting in a rather small number of overall sketch samples, but ML approaches usually require a larger data set as input. In fact, several of the publications presented in our related work section are focused on the stroke-level rather than entire sketches.

Three feature subsets are considered the set of all 165 features, a set of 11 features from related publications from the field of analyzing cognitive assessments, and the combination of both (176 features). The set of 11 features collected from literature that focuses on the evaluation of cognitive performance (Werner et al., 2006; Cohen et al., 2014; Davis et al., 2015), and includes number of strokes, sketching time, stroke distance, duration, average pressure, average velocity, variation of velocity, number of pauses, average pause duration, the ratio between sketching and pausing, and average lift duration. The complete list of features is provided in the Appendix section.

The set of experiments conducted here is concerned with the binary classification of samples as healthy or suspicious, on a combination of sketch-level vs. stroke-level features, the three feature subsets, and the same ten ML methods from above experiments. This results in a total of 60 experiment runs, where each classifier is trained individually on each of the data and feature sets in a 10-fold CV scheme. The selection of classifiers includes linear SVMs, Gaussian SVMs, Logistic Regression, Nearest Neighbors, Naive Bayes, Decision Trees, Random Forests, AdaBoost, Gradient Boosted Trees and Deep Learning. Simple iterative hyperparameter optimization is employed to find optimal settings for each of the ML classifiers.

In addition, AutoML experiments are conducted to verify whether the prediction accuracy can be improved for this problem as well. As in the previous experiment the Google AutoML Tables framework is used to conduct the experiment. Unfortunately, the approach requires at least 1,000 rows of data, which is why only the stroke-level conditions are considered for each of the feature subsets. Automated feature selection is used as part of the AutoML experiments.

### 4.2. Results

In total 152 samples were annotated by experts as healthy and 84 as suspicious [14/40 CDT samples (35.0%), 14/80 TMT samples (17.5%), and 56/116 ROCF samples (48.3%)]. The data was collected from 40 elderly subjects, who participated in a study at a geriatrics daycare clinic. More details on the subjects and the collection method are provided in Section 3.2—CDT, TMT, and ROCF Data Collection Method on page 10. Dividing the data set into labeled strokes results in a total of 6,893 strokes [508/1,211 CDT (41.9%), 117/438 TMT (26.7%), and 2,175/5,244 ROCF (41.5%)].

The most accurate top 5 ML methods for the classification of cognitive test performance are summarized in Table 2. A differentiation is made between sketch-based and stroke-based calculation of features. Sketch-176 includes all 165 sketch-based digital pen features, plus the 11 features related to cognitive testing, whereas Sketch-165 only includes all 165 features and Sketch-11 only includes the cognitive features. The Stroke-176, Stroke-165, and Stroke-11 are their equivalent stroke-based feature subsets. All reported measures are averaged as part of the applied 10-fold cross-validation scheme.

**Table 2:** Top 5 ML methods for cognitive test performance classification per feature subset.

**Feature subset**	**ML method**	**Accuracy**	**Precision**	**Recall**	**F1 score**	**AUC (ROC)**
Sketch-176	Random forest	**85.4%**	**85.5%**	82.1%	0.833	0.821
	SVM (Linear)	**85.4%**	83.9%	86.1%	0.846	0.861
	Gradient boosted Tree	83.3%	83.7%	79.1%	0.806	0.791
	AdaBoost	81.2%	79.7%	78.8%	0.792	0.788
	SVM (Gaussian RBF)	81.2%	81.9%	76.2%	0.778	0.762
Sketch-165	AdaBoost	**87.5%**	**86.0%**	**87.7%**	**0.867**	**0.877**
	SVM (Linear)	**85.4%**	83.9%	86.1%	0.846	**0.861**
	Gradient boosted tree	83.3%	83.7%	79.1%	0.806	0.791
	Random forest	83.3%	82.4%	80.5%	0.812	0.805
	SVM (Gaussian RBF)	81.2%	81.9%	76.2%	0.778	0.762
Sketch-11	Deep learning	77.1%	75.4%	73.0%	0.738	0.730
	Random forest	77.1%	75.4%	73.0%	0.738	0.730
	AdaBoost	72.9%	71.9%	73.7%	0.719	0.737
	SVM (Gaussian RBF)	72.9%	71.8%	65.7%	0.665	0.657
	Decision tree	70.8%	71.5%	73.4%	0.704	0.734
Stroke-176	SVM (Gaussian RBF)	65.0%	64.5%	59.8%	0.588	0.598
	Random forest	63.7%	62.2%	59.2%	0.586	0.592
	Gradient boosted tree	63.5%	62.2%	58.3%	0.571	0.583
	AdaBoost	61.6%	59.5%	56.1%	0.542	0.561
	Logistic regression	61.5%	59.1%	57.0%	0.563	0.570
Stroke-165	SVM (Gaussian RBF)	64.8%	64.4%	59.6%	0.585	0.596
	Gradient boosted Tree	63.8%	62.5%	58.9%	0.579	0.589
	Random forest	63.7%	62.5%	58.5%	0.573	0.585
	AdaBoost	61.8%	59.8%	56.1%	0.541	0.561
	SVM (Linear)	61.4%	59.0%	56.8%	0.560	0.568
Stroke-11	SVM (Linear)	61.6%	59.5%	55.7%	0.534	0.557
	Logistic regression	61.3%	59.1%	55.6%	0.535	0.556
	Deep learning	61.3%	59.2%	55.1%	0.523	0.551
	Gradient boosted Tree	61.2%	58.7%	55.9%	0.544	0.559
	SVM (Gaussian RBF)	61.2%	59.3%	54.6%	0.511	0.546

*The reported measures are average values (10-CV). Best results are highlighted as bold*.

Employing iterative hyperparameter optimization for each of the ML classifiers results in the following model configurations. The SVMs are set to a *C* of 1.5 with a maximum number of iterations of 10,000. Logistic Regression is set to a *C* of 8.0 with *newton-cg* as solver. A 7-Nearest Neighbors approach is used, and Naive Bayes is set to a smoothing value of 10^−9^. All decision tree based approaches are left at default settings, only the learning rate of AdaBoost is set to 0.5. The Deep Learning network is set to 1,000 hidden layers with an *alpha* of 1, a learning rate of 0.001, *Adam* as solver and *tanh* activation function.

**Figure 8:**
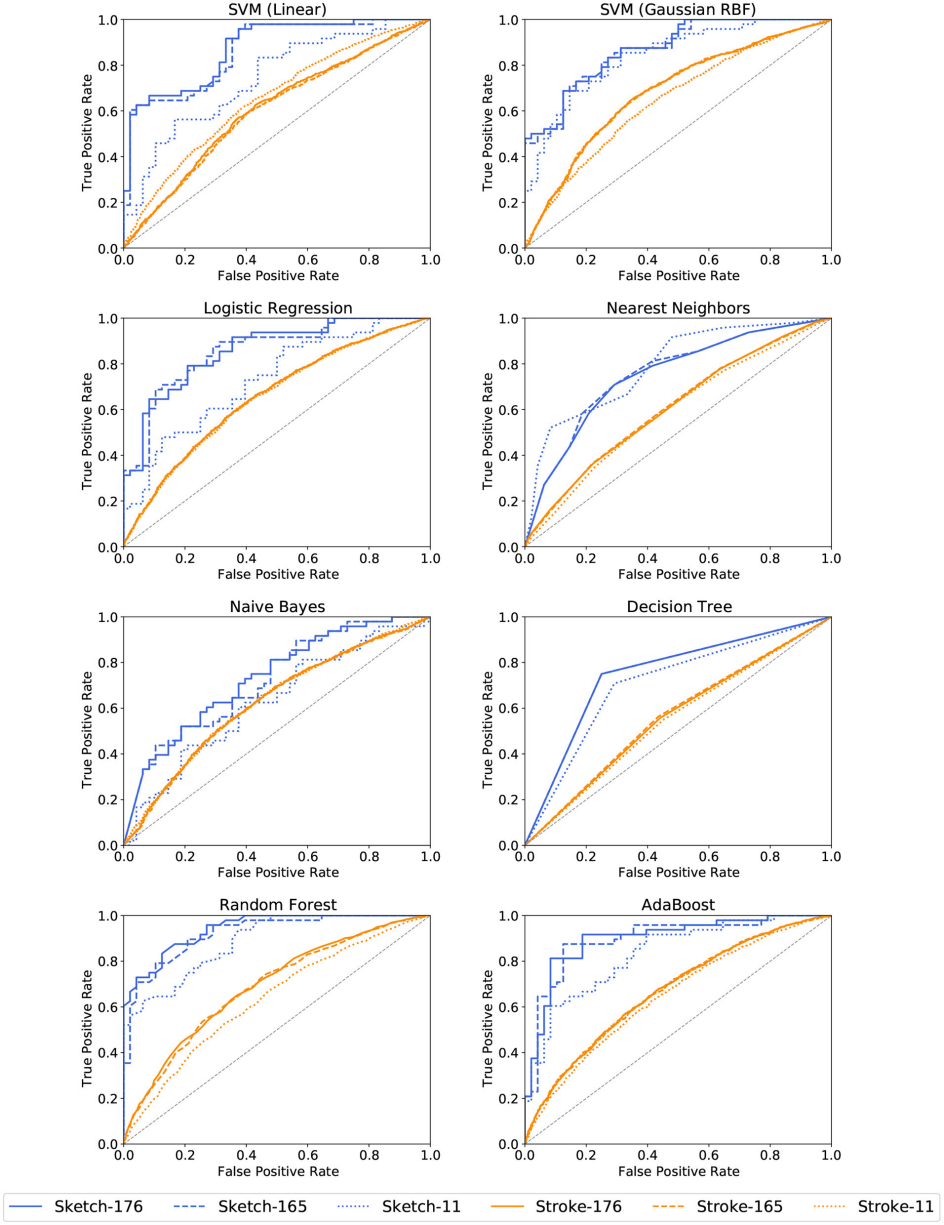
ROC curves for cognitive test performance classification per ML method and feature subset. The jagged curves are a result of the binary classification.

**Figure 9:**
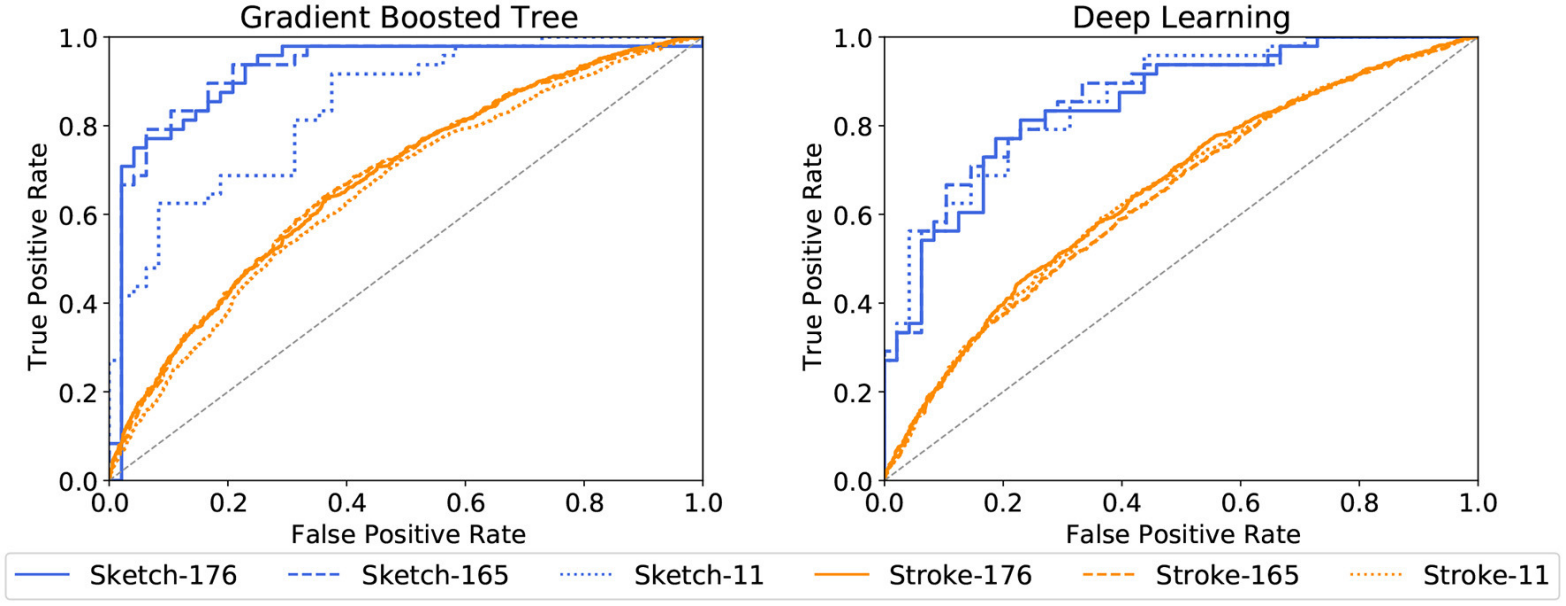
ROC curves for cognitive test performance classification per ML method and feature subset (continued).

AdaBoost achieves the highest accuracy with 87.5% on the set of all 165 sketch-based features, followed by the linear SVM with 85.4%. A similar accuracy of 85.4% is achieved by Random Forests and the linear SVMs on the set of 176 features. Considering only the 11 features related to cognitive testing, the prediction accuracies drop below 80% with the highest accuracy achieved by the Deep Learning approach with 77.1%. Stroke-based approaches stay above the chance level with a maximum of 65.0% recognition accuracy for Gaussian RBF SVMs for all 176 features and 64.8% for the 165 features set. The corresponding ROC curves in Figures 8, 9 support this observation. All stroke-based approaches produce a flat ROC curve with a maximum AUC of 0.598 for the Gaussian RBF SVMs. In contrast, the ROC curves of their sketch-based equivalents are much steeper with the highest AUC achieved by the AdaBoost approach with a value of 0.877, followed by 0.861 for the linear SVMs.

The highest precision and recall scores are achieved by AdaBoost as well with 86.0% and 87.7% respectively. In general, the sketch-based feature sets with 165 and 176 features perform best, and stroke-level feature approaches are just above chance level. Similarly, the F1 scores are above 0.8 for the bigger sketch-level feature sets, which indicates a good balance between precision and recall. In contrast, the F1 scores for the stroke-level conditions are all below 0.6.

**Figure 10:**
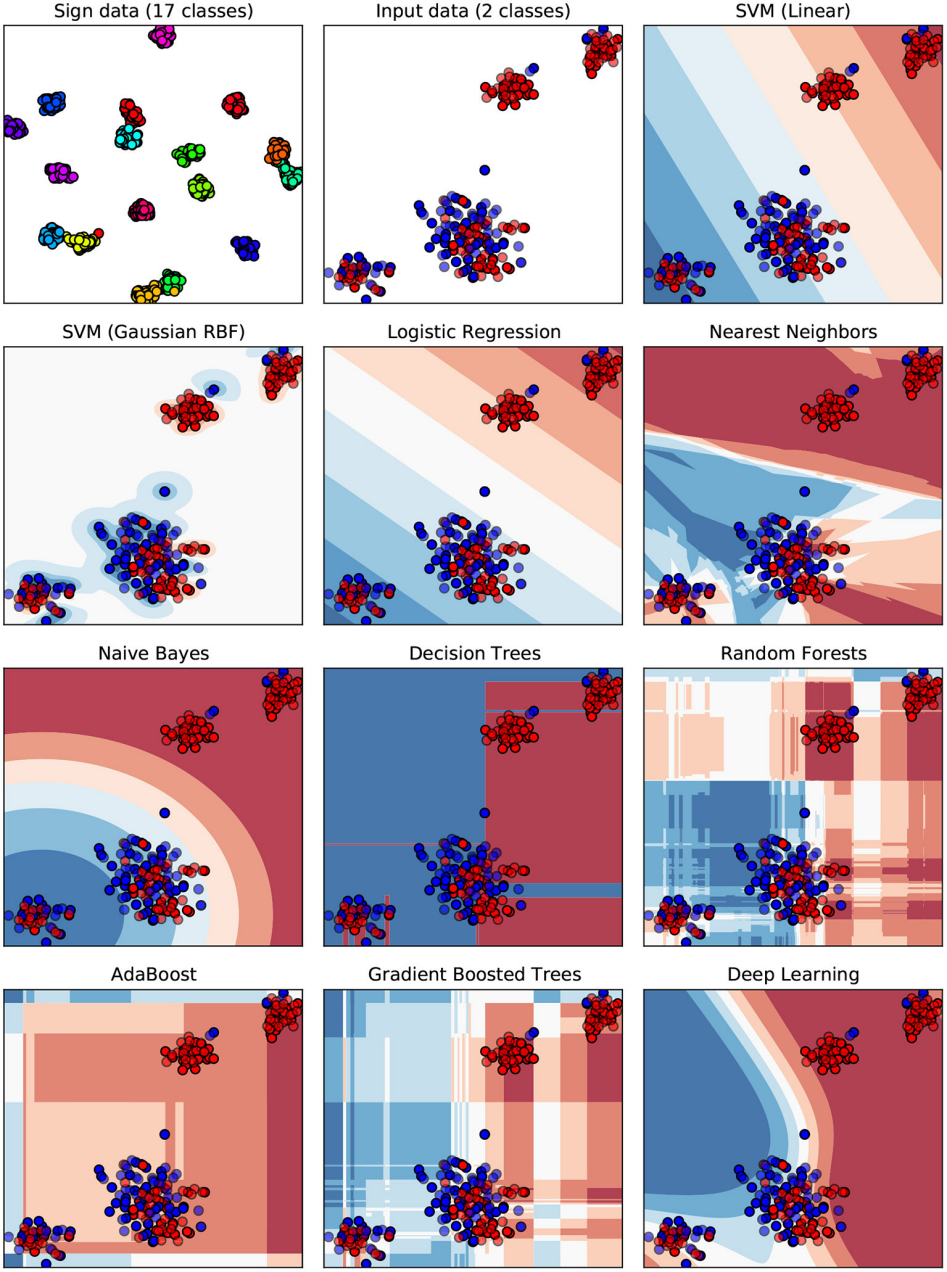
A visualization of decision boundaries for the ML classifiers. The feature space of 176 dimensions (features) is mapped to the 2d Euclidean space using t-SNE dimensionality reduction. Each dot represents the input feature vector of one sample from the data set. Classes are color-coded (red = healthy, blue = suspicious), solid points are training samples, while testing samples are semi-transparent. The Sign data set (Almaksour et al., 2010) with its 17 classes is included as reference, to give an example of an easily separable data set.

To help understand why the results are rather poor, a visualization of the underlying decision boundaries for the individual ML classifiers is provided in Figure 10. The t-distributed stochastic neighbor embedding (t-SNE) algorithm presented by Van Der Maaten and Hinton (2008) and Van Der Maaten (2014) is used to map the high-dimensional feature space of 165 dimensions (the digital pen features) down to the two-dimensional Euclidean space. In data sciences and ML, t-SNE is commonly used as a nonlinear dimensionality reduction technique, which is well-suited for embedding high-dimensional data for visualization in a low-dimensional space of two dimensions. Using standard techniques such as principal component analysis (PCA) or multi-dimensional scaling for dimensionality reduction results in crowded plots, where many data points fall close together in the mapped two-dimensional space, resulting in unhelpful layouts (Eitz et al., 2012). In contrast, t-SNE specifically addresses this problem by computing a mapping of distances in high-dimensional space to distances in low-dimensional space such that smaller pairwise distances in high-dimensional space (which would produce the crowding problem) are mapped to larger distances in two-dimensional space, while still preserving overall global distances (Eitz et al., 2012). The first two plots of Figure 10 show the raw digital pen feature vectors for each sketch after being mapped using t-SNE (*components* set to 2, a *perplexity* of 30, *early exaggeration* set to 12.0 and a *learning rate* of 200.0 with a maximum of 1,000 iterations). Different classes are indicated by color (red = healthy, blue = suspicious), solid points are training samples, whereas semi-transparent points are used for testing. The Sign data set (Almaksour et al., 2010) is included as a reference, because research shows that its classes can be easily separated by ML classifiers. The corresponding plot shows 17 almost completely separated clusters, one for each of the classes of the data set. All remaining plots of Figure 10 show the decision boundaries of the ML classifiers for the prediction of cognitive test performance.

**Figure 11:**
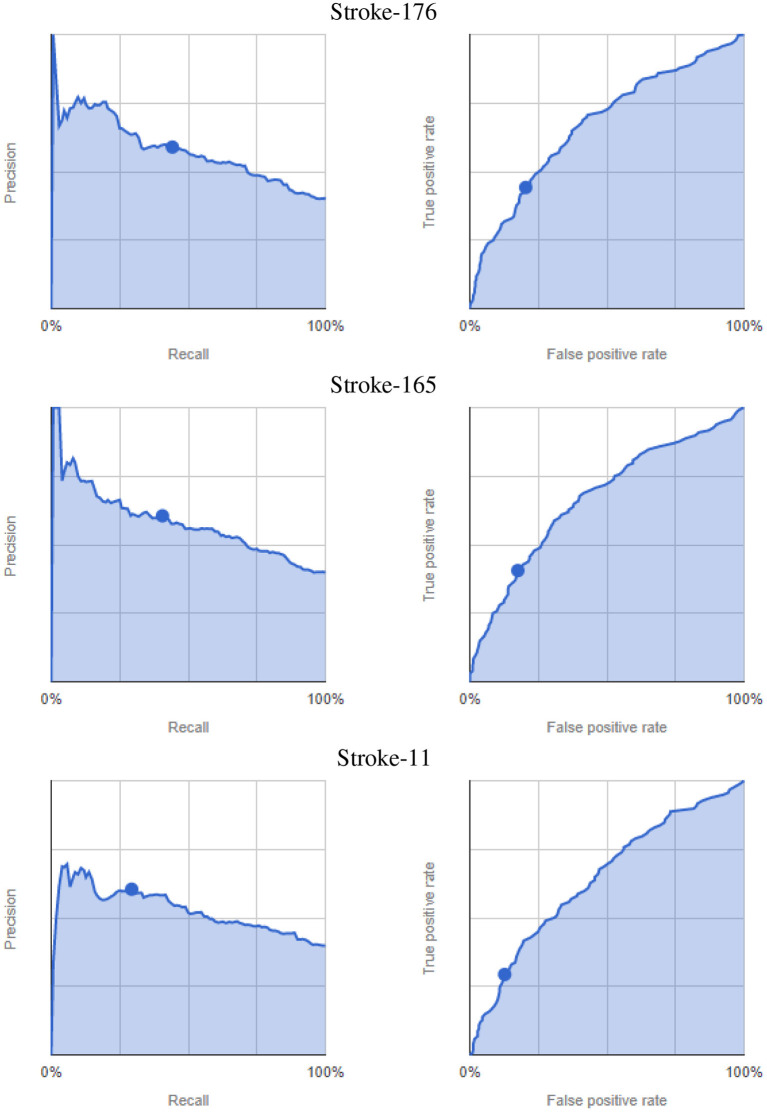
AutoML-based classification of cognitive test performance per feature subsets. Precision-recall curves (left) and ROC curves (right), dots indicate the default 0.5 score threshold set by the framework.

The results of the AutoML experiment on the stroke-level feature sets are summarized in Table 3, with the corresponding precision-recall and ROC curves presented in Figure 11. Due to the binary classification, the framework does not provide additional precision-recall score thresholds like for the previous AutoML experiment. The results show only a minor improvement in accuracy of 65.8%, with comparably high log loss and low precision, recall and F1 score. Similarly, the ROC curves are rather flat with the highest AUC ROC value of 0.671.

**Table 3:** Results of the AutoML experiments for cognitive test performance classification with different feature subsets used for training.

**Feature subset**	**Accuracy**	**F1 score**	**Log loss**	**Precision**	**Recall**	**AUC (PR)**	**AUC (ROC)**
Stroke-176	65.5%	0.735	0.630	68.3%	79.6%	0.581	0.671
Stroke-165	65.8%	0.486	0.631	60.5%	40.6%	0.582	0.668
Stroke-11	64.2%	0.395	0.646	60.4%	29.3%	0.527	0.641

*Data split: 80% training, 10% validation and 10% testing. Only stroke-based features are considered, because the AutoML approach requires at least 1,000 rows of data*.

### 4.3. Discussion

Interestingly, the AdaBoost approach works best on this classification problem, whereas its results on the sketch recognition problems in previous experiments were rather unremarkable. It is suspected that this is due to two circumstances. First of all, the here considered problem is a binary classification problem instead of the multi-class classification in the previous experiments. In addition, a simple fine-tuning of the AdaBoost hyperparameters drastically improved the prediction accuracy. An accuracy of 87.5% with similar precision and recall values is not perfect, but it outperforms all previous approaches on the cognitive tests considered (Schrter et al., 2003; Werner et al., 2006; Cohen et al., 2014; Davis et al., 2015).

**Figure 12:**
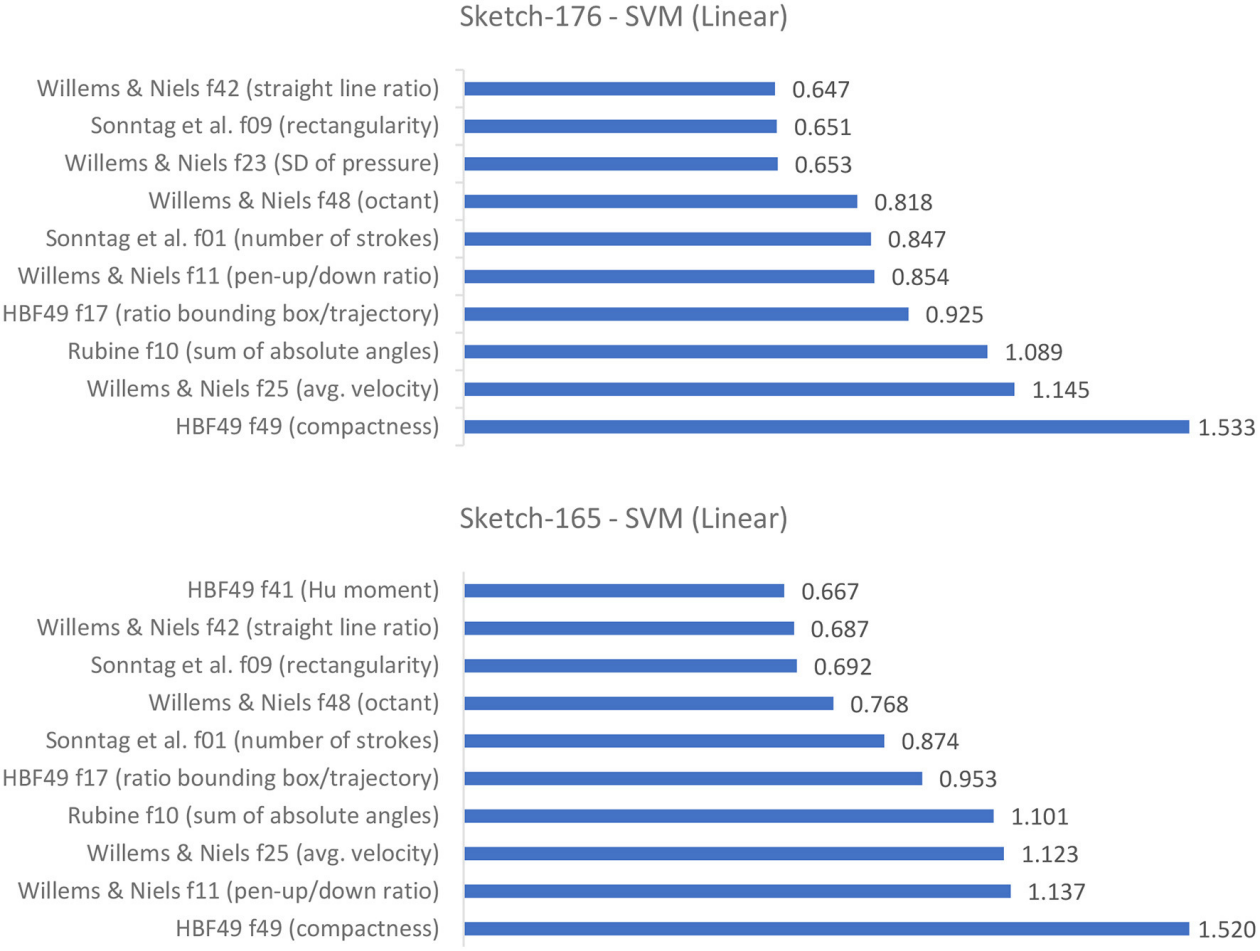
Average weights assigned to the input features of the linear SVMs (top 10).

It seems that the 11 additional features for cognitive assessments are not providing any added value. The best results are achieved on the here presented set of 165 digital pen features. An interesting observation is, that the SVMs and the Gradient Boosted Tree models seem to have found an optimum on their own for both Sketch-176 and Sketch-165, as the measurement results are the same in all six cases, only the ROC curves differ slightly. Figure 12 shows the top 10 averaged feature weights for the linear SVMs. All ten most important features are similar between both feature sets, only their weights differ. This might indicate that the classifiers do select more relevant features on their own during training and that providing the highest possible amount of features can be a good choice. Further investigation into this direction might be necessary to confirm or dismiss this hypothesis. Judging from the importance of features in Figure 12, the HBF49 feature *compactness* and Willems and Niels feature *average velocity* are among the most important decision factors. Compactness models how close the sample points are to each other, which together with the average velocity leads to the assumption that cognitively impaired subjects tend to sketch noticeably slower than their healthy counterparts. This finding is supported by related work, which shows that patients with cognitive impairment experience loss of fine motor performance and that temporal measurements are higher in the cognitively impaired groups (Schrter et al., 2003; Werner et al., 2006). However, analyzing Figure 12 it is clear that the other top 10 features likewise have a high influence. Hence, it is not enough to look exclusively at the temporal features.

A closer look at Figure 10 gives an intuition on the reason why the ML classifiers were able to achieve higher accuracies in previous experiments. Based on the visualization of the samples' feature vectors the reference Sign data set is separated more easily in the high dimensional space than is the case for the cognitive assessments data. Some of the healthy samples form clusters in the upper right, while the majority of points is intermixed in the middle and lower left, making it difficult for several classifiers to divide the data appropriately. This might be an oversimplification of these complex models, but it nevertheless provides some perspective on how the models work internally.

Considering both the ten off-the-shelf ML classifiers as well as AutoML, it is clear that the stroke-level approach does not work in this setting. This could be due to several reasons. Firstly, not all of the digital pen features are necessarily well defined on single strokes, e.g., average lift duration, pen-up/pen-down ratio or straight-line ratio. Secondly, it is possible that the effects of cognitive impairment do not show equally well in all types of pen strokes, or that the features are not sensitive enough to detect this information from as little input as single strokes. Also, it might be necessary to include a more fine-grained annotation of the data. Currently, entire sketches are labeled as healthy or suspicious based on the scoring result of the entire sketch. For the stroke labeling, this sketch-wise annotation is propagated to each individual stroke. Instead, it might be more sensible to annotate individual strokes which are part of a specific error made by the subject.

## 5. Conclusion and Future Work

This work demonstrated how digital pen features, such as geometrical, spacial, temporal and pressure characteristics can be used to model users' cognitive performance. We showed how such features can be computed by using commercially available digital pen technology in a real world scenario, and how to effectively analyze them in real-time using machine learning methods. Two approaches showed the utility of digital pen features for the analysis of paper-pencil-based neurocognitive assessments in the medical domain. A traditional approach showed how cognitive assessments can be analyzed as part of an interactive cognitive assessment tool using content analysis and medical scoring schemes, thereby reducing manual scoring effort and producing unbiased, explainable results. A second, innovative approach showed how cognitive test performance can be predicted by only looking at the sketch characteristics modeled by the digital pen features, without performing further semantic content analysis. Using standard ML techniques, the set of 176 features outperformed all previous approaches on the cognitive tests considered, i.e., the Clock Drawing Test, the Rey-Osterrieth Complex Figure Test, and the Trail Making Test. It automatically scored cognitive tests with up to 87.5% accuracy in the binary classification task of categorizing sketches as healthy or suspicious. This supports more automatic, more objective and accurate diagnostics of pen sensor input, which can be used in hospitals and retirement homes to transparently evaluate cognitive performance (i.e., without explicit testing), to guide medical interventions, and to adapt cognitive training in a personalized manner.

The successful deployment of our interactive cognitive assessment tool in a geriatrics daycare clinic shows that digital pen technologies are compatible with real world settings. Writing and pen interaction also constitute familiar work practice in hospitals, so the introduction of digital pen technology is less likely to disrupt everyday processes, compared to other alternatives. In contrast to other automated cognitive assessment tools we do not necessarily have to perform any content analysis, but instead are able to only consider how something was sketched, which makes our approach independent of the specific test. In addition, we create a causal link between user behavior, pen features and test result, thereby improving the explainability of our machine learning models. These findings are also significant because they enable predictive analytics based on pen input and open up opportunities to design new cognitive testing capabilities, as well as adapting cognitive training in a personalized manner based on individual data.

More generally, our approach can be used in various intelligent user interface frameworks to evaluate and improve multimodal human-computer interaction through pen-based analytic capabilities in domains such as healthcare and education. Future work could evaluate whether this approach can be used to evaluate and improve multimodal human-computer interaction through pen-based analytic capabilities in different domains (Oviatt, 2013; Oviatt et al., 2017). For example, some approaches show how digital pen features can aid in predicting math expertise (Oviatt and Cohen, 2014; Zhou et al., 2014), task difficulty and user performance (Barz et al., 2020). This includes analyzing which features have the highest impact on the recognition results, thereby producing more transparent recognition results. Explanatory interactive machine learning (IML) (Teso and Kersting, 2019) could be used to create interfaces which provide direct feedback to the user and allow for the inclusion of expert knowledge into the ML process (human-in-the-loop). A prototypic example of such a system for sketch recognition was presented as part of the MIT Media Lab course on IML^2^ using the Gesture Recognition Toolkit (Gillian and Paradiso, 2014).

Another promising topic that is currently being investigated is to link the digital pen features directly to the individual scoring results. Together with our colleagues at the Charit in Berlin we aim to further evaluate this aspect as part of an interactive user interface. The transparent feedback about which features contribute to which item in the scoring result helps physicians to understand why the model makes certain predictions and can therefore increase trust in the system. It might also help physicians to discover new approaches for the analysis of cognitive assessments. Furthermore, by taking into account the knowledge of domain experts as part of an IML system, trust and explainability of the underlying ML models could be further improved.

## Data Availability Statement

The raw data supporting the conclusions of this article will be made available by the authors, without undue reservation.

## Ethics Statement

The studies involving human participants were reviewed and approved by Charité-Universitätsmedizin Berlin, Berlin, Germany. The patients/participants provided their written informed consent to participate in this study.

## Author Contributions

All authors listed have made a substantial, direct, and intellectual contribution to the work and approved it for publication.

## Funding

This research was part of the Intera-KT project (BMBF, 16SV7768). The research has also been supported by the GeAR project (BMBF, 01JD1811C), and the Endowed Chair of Applied Artificial Intelligence, Oldenburg University.

## Conflict of Interest

The authors declare that the research was conducted in the absence of any commercial or financial relationships that could be construed as a potential conflict of interest.

## Publisher's Note

All claims expressed in this article are solely those of the authors and do not necessarily represent those of their affiliated organizations, or those of the publisher, the editors and the reviewers. Any product that may be evaluated in this article, or claim that may be made by its manufacturer, is not guaranteed or endorsed by the publisher.

## Footnotes

^1^ https://www.neosmartpen.com

^2^ https://vimeo.com/76839534
