# Supplementary material for: Modeling Users' Cognitive Performance Using Digital Pen Features
Source: Front Artif Intell. 2022 May 3;5:787179. doi: 10.3389/frai.2022.787179 (PMC9113515; doi:10.3389/frai.2022.787179)
Supplement: Supplementary file 1 [file Data_Sheet_1.pdf]

1 APPENDIX

1.1 List of Digital Pen Features

In this section we provide the complete list of features from the various feature sets that we consider. For formal mathematical definitions please refer to the original publications. Please note that the feature sets are not necessarily distinct.

Our reference implementation is made publicly available on GitHub at:
`https://github.com/DFKI-Interactive-Machine-Learning/ink-features`

1.1.1 Rubine's Feature Set

These are the 13 features described by Rubine (Rubine, 1991):

1. Cosine of initial angle
2. Sine of initial angle
3. Length of bounding box diagonal
4. Angle of the bounding box diagonal
5. Distance between first and last point
6. Cosine of the angle between first and last point
7. Sine of the angle between first and last point
8. Total gesture length
9. Total angle traversed
10. Sum of the absolute value of the angle at each point
11. Sum of the squared value of the angle at each point
12. Maximum speed (squared) of the gesture
13. Duration of the gesture

1.1.2 Willems & Niels Feature Set

The feature set described by Willems and Niels (Willems and Niels, 2008) contains the following 89 features:

1. Length of the gesture
2. Area
3. Compactness
4. Eccentricity
5. Ratio between co-ordinate axes
6. Closure
7. Circular variance
8. Curvature
9. Average curvature
10. Standard deviation in curvature
11. Pen up/down ratio
12. Average direction

13. Perpendicularity
14. Average perpendicularity
15. Standard deviation in perpendicularity
16. Centroid offset
17. Length of first principal axis
18. Sine orientation of principal axis
19. Cosine orientation of principal axis
20. Rectangularity
21. Maximum angular difference
22. Average pressure
23. Standard deviation of pressure
24. Duration
25. Average velocity
26. Standard deviation of velocity
27. Maximum velocity
28. Average acceleration
29. Standard deviation of acceleration
30. Maximum acceleration
31. Minimum acceleration
32. Number of cups
33. Offset of the first cup
34. Offset of the last cup
35. Initial horizontal offset
36. Final horizontal offset
37. Initial vertical offset
38. Final vertical offset
39. Number of straight lines
40. Average length of straight lines
41. Standard deviation of straight line length
42. Straight line ratio
43. Largest straight line ratio
44. Number of pen down events
45. Octants 1
46. Octants 2
47. Octants 3
48. Octants 4
49. Octants 5
50. Octants 6

51. Octants 7
52. Octants 8
53. Number of connecting strokes
54. Number of crossings
55. Cosine of initial angle
56. Sine of initial angle
57. Length of the bounding box diagonal
58. Angle of the bounding box diagonal
59. Length between first and last point
60. Cosine of first to last point
61. Sine of first to last point
62. Absolute curvature
63. Squared curvature
64. Macro perpendicularity
65. Average macro perpendicularity
66. Standard deviation in macro perpendicularity
67. Ratio of principal axes
68. Average centroidal radius
69. Standard deviation of the centroidal radius
70. Sin chain code 1
71. Cos chain code 1
72. Sin chain code 2
73. Cos chain code 2
74. Sin chain code 3
75. Cos chain code 3
76. Sin chain code 4
77. Cos chain code 4
78. Sin chain code 5
79. Cos chain code 5
80. Sin chain code 6
81. Cos chain code 6
82. Sin chain code 7
83. Cos chain code 7
84. Sin chain code 8
85. Cos chain code 8
86. Average stroke length
87. Standard deviation in stroke length
88. Average stroke direction

89. Standard deviation in stroke direction

1.1.3 HBF49 Feature Set

The HBF49 feature set (Delaye and Anquetil, 2013) contains the following 49 features:

1. Horizontal position of first point
2. Vertical position of first point
3. Horizontal position of last point
4. Vertical position of last point
5. First point to last point vector length
6. Sine of first point to last point vector
7. Cosine of first point to last point vector
8. Closure
9. Sine of initial angle
10. Cosine of initial angle
11. Horizontal inflexion
12. Vertical inflexion
13. Downstroke proportion
14. Number of strokes
15. Angle of the bounding box diagonal
16. Trajectory length
17. Ratio between bounding box and trajectory length
18. Deviation
19. Average direction
20. Curvature
21. Perpendicularity
22. k-Perpendicularity
23. k-Angle
24. Dominant direction 1
25. Dominant direction 2
26. Dominant direction 3
27. Dominant direction 4
28. Local changes in direction 1
29. Local changes in direction 2
30. Local changes in direction 3
31. Local changes in direction 4
32. 2D histogram 1
33. 2D histogram 2
34. 2D histogram 3

35. 2D histogram 4
36. 2D histogram 5
37. 2D histogram 6
38. 2D histogram 7
39. 2D histogram 8
40. 2D histogram 9
41. Hu moment 1
42. Hu moment 2
43. Hu moment 3
44. Hu moment 4
45. Hu moment 5
46. Hu moment 6
47. Hu moment 7
48. Normalized convex hull area
49. Compactness

1.1.4 Sonntag et al. Feature Set

The 14 features described by Sonntag et al. (Sonntag et al., 2014) are:

1. Number of Strokes
2. Length
3. Area
4. Perimeter Length
5. Compactness
6. Eccentricity
7. Principal Axes
8. Circular Variance
9. Rectangularity
10. Closure
11. Curvature
12. Perpendicularity
13. Signed Perpendicularity
14. Angles after Equidistant Resampling

1.1.5 Cognitive Performance Feature Set

The set of 11 features defined in literature that focuses on the evaluation of cognitive performance (Cohen et al., 2014; Davis et al., 2015; Werner et al., 2006) consists of:

1. Number of strokes
2. Sketching time

3. Stroke distance
4. Duration
5. Average pressure
6. Average velocity
7. Variation of velocity
8. Number of pauses
9. Average pause duration
10. Ratio between sketching and pausing
11. Average lift duration

REFERENCES

Cohen, J., Penney, D., Davis, R., Libon, D., Swenson, R., Ajilore, O., et al. (2014). Digital clock drawing: Differentiating “thinking” versus “doing” in younger and older adults with depression. *Journal of the International Neuropsychological Society* 20, 920–928. doi:10.1017/S1355617714000757

Davis, R., Libon, D., Au, R., Pitman, D., and Penney, D. (2015). THink: Inferring Cognitive Status from Subtle Behaviors. vol. 36, 49–60. doi:10.1609/aimag.v36i3.2602

Delaye, A. and Anquetil, E. (2013). HBF49 feature set: A first unified baseline for online symbol recognition. *Pattern Recognition* 46, 117–130. doi:10.1016/j.patcog.2012.07.015

Rubine, D. (1991). Specifying gestures by example. In *Proceedings of the 18th Annual Conference on Computer Graphics and Interactive Techniques* (New York, NY, USA: ACM), SIGGRAPH ’91, 329–337. doi:10.1145/122718.122753

Sonntag, D., Weber, M., Cavallaro, A., and Hammon, M. (2014). Integrating digital pens in breast imaging for instant knowledge acquisition. *AI Magazine* 35, 26–37. doi:10.1609/aimag.v35i1.2501

Werner, P., Rosenblum, S., Bar-On, G., Heinik, J., and Korczyn, A. (2006). Handwriting process variables discriminating mild alzheimer’s disease and mild cognitive impairment. *The Journals of Gerontology Series B: Psychological Sciences and Social Sciences* 61, P228–P236. doi:10.1093/geronb/61.4.p228

Willems, D. and Niels, R. (2008). *Definitions for Features used in Online Pen Gesture Recognition*. Tech. rep., NICI, Radboud University Nijmegen
